# Structural mechanisms of human sodium-coupled high-affinity choline transporter CHT1

**DOI:** 10.1038/s41421-024-00731-7

**Published:** 2024-11-26

**Authors:** Jing Xue, Hongwen Chen, Yong Wang, Youxing Jiang

**Affiliations:** 1 Institute of Aging & Tissue Regeneration, Renji Hospital, Shanghai Jiao Tong University School of Medicine, Shanghai, China; 2 Department of Molecular Genetics, University of Texas Southwestern Medical Center, Dallas, TX USA; 3 College of Life Sciences, Zhejiang University, Hangzhou, Zhejiang China; 4 Howard Hughes Medical Institute and Department of Physiology, University of Texas Southwestern Medical Center, Dallas, TX USA; 5 Department of Biophysics, University of Texas Southwestern Medical Center, Dallas, TX USA

**Keywords:** Cryoelectron microscopy, Molecular biology

## Abstract

Mammalian sodium-coupled high-affinity choline transporter CHT1 uptakes choline in cholinergic neurons for acetylcholine synthesis and plays a critical role in cholinergic neurotransmission. Here, we present the high-resolution cryo-EM structures of human CHT1 in apo, substrate- and ion-bound, hemicholinium-3-inhibited, and ML352-inhibited states. These structures represent three distinct conformational states, elucidating the structural basis of the CHT1-mediated choline uptake mechanism. Three ion-binding sites, two for Na^+^ and one for Cl^–^, are unambiguously defined in the structures, demonstrating that both ions are indispensable cofactors for high-affinity choline-binding and are likely transported together with the substrate in a 2:1:1 stoichiometry. The two inhibitor-bound CHT1 structures reveal two distinct inhibitory mechanisms and provide a potential structural platform for designing therapeutic drugs to manipulate cholinergic neuron activity. Combined with the functional analysis, this study provides a comprehensive view of the structural mechanisms underlying substrate specificity, substrate/ion co-transport, and drug inhibition of a physiologically important symporter.

## Introduction

Choline is an essential nutrient for the development and function of the brain^1–3^. In cholinergic neurons, choline is the precursor of acetylcholine (ACh), a vital neurotransmitter for muscle control, autonomic nervous system function, and multiple cognitive activities^4–6^. During neurotransmission, ACh is released from the presynaptic neurons into the synaptic cleft to activate postsynaptic ACh receptors before being hydrolyzed to acetate and choline^7–10^. Subsequently, choline is reuptake into the presynaptic neurons for ACh synthesis by the sodium-coupled high-affinity choline transporter CHT1 expressed in the cholinergic neurons and localized in the presynaptic terminal^11–16^. *CHT1*-knockout mice are immobile with respiratory failure and fail to survive the first few hours of life^17,18^. Dysfunctions of CHT1 in humans are associated with distal hereditary motor neuropathy^19–21^, congenital myasthenic syndrome^22–24^, and Alzheimer’s disease^6,25,26^, highlighting its critical role in cholinergic neurotransmission.

CHT1 belongs to the mammalian solute carrier family SLC5 which also includes Na^+^/glucose cotransporters (SGLTs) and is classified as SLC5A7^14,27^. CHT1 functions as a symporter that utilizes the chemical gradient of Na^+^ ions across the plasma membrane to drive choline uptake^13,16,28,29^. However, the co-transport stoichiometry between Na^+^ and choline in CHT1 has not been clearly defined. In addition, the CHT1 transport function is also Cl^–^-dependent^13,28^, and whether Cl^–^ plays a regulatory role or is co-transported with the substrate remains elusive. As a rate-limiting determinant of acetylcholine synthesis, inhibiting CHT1-mediated choline uptake can reduce cholinergic transmission function^14,16,29^. Several small-molecule CHT1 inhibitors have been developed to provide specific tools for manipulating the cholinergic neuron activity, including the competitive inhibitor hemicholinium-3 (HC3) and the noncompetitive inhibitor ML352^13,30,31^.

Despite the physiological significance of the CHT1 transporter, little is known about the structural basis of its substrate recognition, ion binding, transport mechanism, and drug inhibition. Here, we present the high-resolution cryogenic electron microscopy (cryo-EM) structures of human CHT1 in apo, substrate- and ion-bound, HC3-inhibited, and ML352-inhibited states, providing a clear visualization of the molecular details of various protein–ligand interactions in CHT1. These structures also represent three distinct conformational states, revealing the structural basis of the CHT1-mediated choline uptake mechanism. Furthermore, the structural insight into CHT1 inhibition also provides a potential platform for therapeutic drug design.

## Results

### Functional characterization of CHT1

**Fig. 1:**
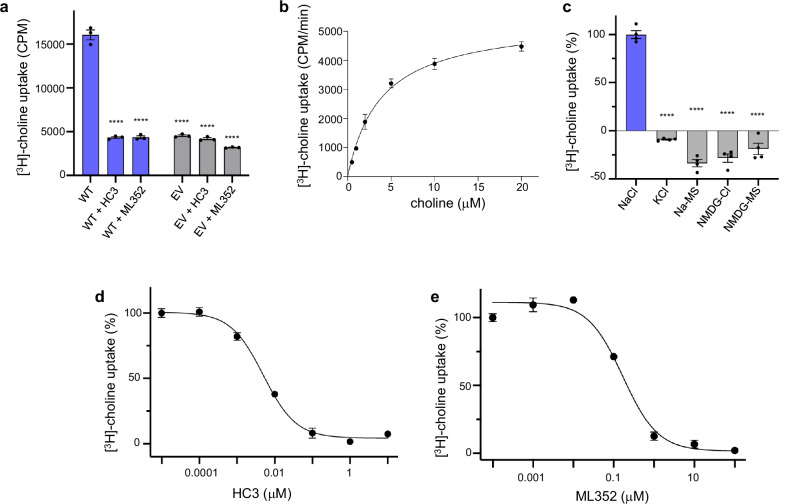
Radioactive choline uptake assay of human CHT1 expressed in HEK293 cells. **a** Radioactive choline uptake of the CHT1-expressing HEK293 cells (wild-type, blue bar) as compared to the background radioactivity from the control cells transfected with the empty vector (EV, gray bar) 10 min after adding 0.1 mM choline with 10% of [^3^H]-choline. The inhibition of CHT1-mediated choline uptake was measured at 0.1 mM HC3 or 1 mM ML352. Data are mean ± SEM (*n* = 3 independent experiments). One-way ANOVA; ****P ≤ 0.0001. **b** Concentration-dependent choline uptake. Data points are mean ± SEM (*n* = 3 independent experiments) and fitted to the Michaelis–Menten equation with *K*_M_ = 3.86 ± 0.68 mM. **c** Na^+^ and Cl^–^-dependent choline uptake. Data are mean ± SEM (*n* = 4 independent experiments) and are normalized against the radioactivity measurement with NaCl in the reaction solution. One-way ANOVA; ****P ≤ 0.0001. **d**, **e** Concentration-dependent inhibition of CHT1-mediated choline uptake by HC3 (**d**) and ML352 (**e**). Data points are mean ± SEM (*n* = 3 for HC3 and *n* = 3–6 for ML352) and fitted to the three-parameter dose–response curves (GraphPad Prism 9) with IC_50_ of 4.98 ± 1.04 nM for HC3 and 168.6 ± 49.4 nM for ML352.

A choline uptake assay was used to measure the human CHT1 transport activity (see Materials and Methods). In this assay, radioactive [^3^H]-choline substrate was added to the extracellular side of CHT1-expressing HEK293 cells, and the CHT1-mediated choline uptake was monitored by measuring the radioactivity of the cells after adding the substrate. A significantly higher accumulation of radioactivity was observed in CHT1-expressing HEK293 cells as compared to the control cells transfected with the empty vector, manifesting the CHT1-mediated choline influx (Fig. 1a). The linear increase of cell radioactivity indicates a constant rate of substrate transport by CHT1 over the experimental period (Supplementary Fig. S1a). A measurement of the rate of radioactivity accumulation at various choline concentrations yielded a *K*_M_ of ~4 μM for CHT1 (Fig. 1b), consistent with previous studies^12,13,32^. No choline influx was observed if extracellular Na^+^ or Cl^–^ was replaced by other monovalent cations (K^+^ or NMDG^+^) or anion (methanesulfonate) (Fig. 1c), confirming the Na^+^ and Cl^–^ dependence of CHT1-mediated choline uptake. The CHT1-mediated choline influx could be effectively abolished by HC3 and ML352 inhibitors (Fig. 1a) and the concentration-dependent inhibition measurement yielded IC_50_ of about 5 nM and 168 nM, respectively (Fig. 1d, e).

### Structural determination of CHT1

Human CHT1 was over-expressed in HEK293S GnTI- cells and purified in LMNG detergent (Materials and Methods; Supplementary Fig. S1b). Despite its small size (MW ~ 64 kDa) and lack of soluble domains, we were able to determine the single particle cryo-EM structures of CHT1 in the apo state (CHT1^apo^) and complex with choline substrate (CHT1^Chol^), inhibitor HC3 (CHT1^HC^), and inhibitor ML352 (CHT1^ML^) to the resolutions of 2.7 Å, 2.4 Å, 3.7 Å, and 2.9 Å, respectively (Materials and Methods; Supplementary Fig. S2 and Table S1). The EM density maps of these structures are of high quality for accurate model building for the major part of the CHT1 transporter and the bound ligands (Supplementary Fig. S3).

**Fig. 2:**
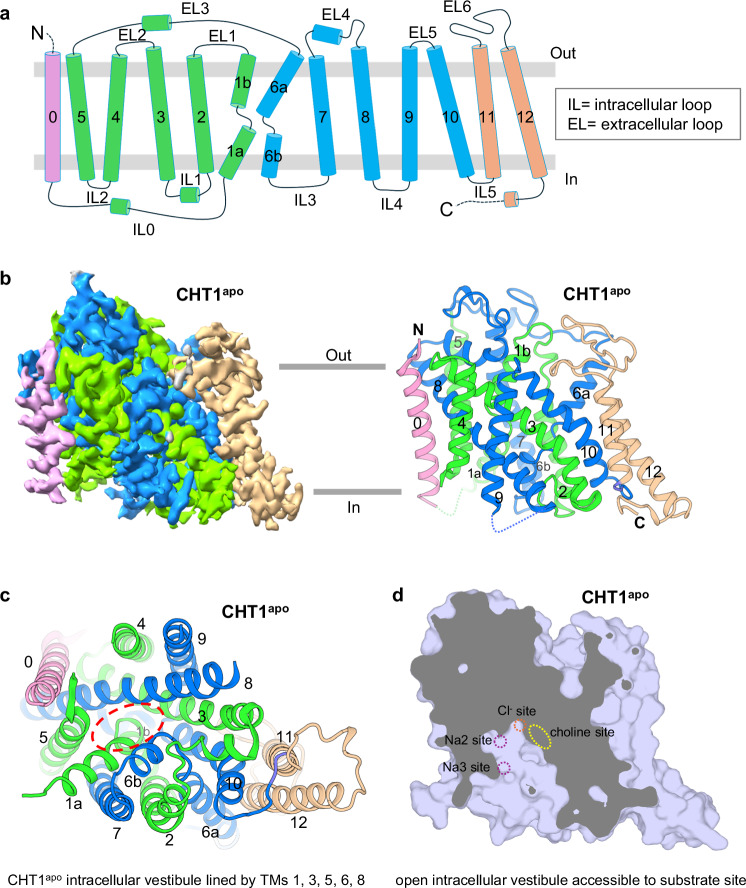
The overall structure of the apo human CHT1 in inward-open conformation. **a** Topology diagram of human CHT1. Segments of TM0, TMs 1–5, TMs 6–10, and TMs 11–12 are individually colored. **b** Side view of 3D reconstruction (left) and cartoon representation of the CHT1^apo^ structure with the four segments individually colored as the topology diagram in **a**. **c** Bottom view of the apo CHT1 from the intracellular side. The red dashed oval marks the entrance of the intracellular vestibule. **d** Side view of the cross-section of the surface-rendered CHT1^apo^ illustrates its inward-open conformation. The dotted circles and oval mark the locations of ions and substrate-binding sites.

The four structures capture the transporter in three different conformations with CHT1^apo^ and CHT1^ML^ in an inward-open state, CHT1^Chol^ in an inward-facing partially open state, and CHT1^HC^ in an outward-open state. CHT1^apo^ will be used here for the initial description of the overall structure and the conformational changes between different states will be further discussed later. CHT1 has an overall architecture similar to that of SGLT transporters (Supplementary Figs. S4 and S5a)^27,33^. It consists of 13 transmembrane (TM) helices (TMs 0–12) with its N-terminus facing the extracellular side and its C-terminus facing the cytoplasmic side (Fig. 2a–c). TMs 1–10 constitute the core of CHT1 with a classical LeuT-fold in which TMs 1–5 and TMs 6–10 form two pseudo-symmetric repeats and TM1 and TM6 are broken into two short helices. The rest of the TM domain (TMs 0, 11, and 12) are positioned at the periphery of the CHT1 core (Fig. 2a–c). The CHT1^apo^ structure adopts an inward-open conformation with a solvent-accessible vestibule extending from the cytosol deep into the substrate-binding pocket at the center of the transporter. The intracellular vestibule is surrounded by charged or hydrophilic residues from the cytosolic halves of TMs 1a, 3, 5, 6b, and 8, providing a passage for the cargo release from the central substrate site (Fig. 2c, d; Supplementary Fig. S5b).

### Substrate and ion binding in CHT1

**Fig. 3:**
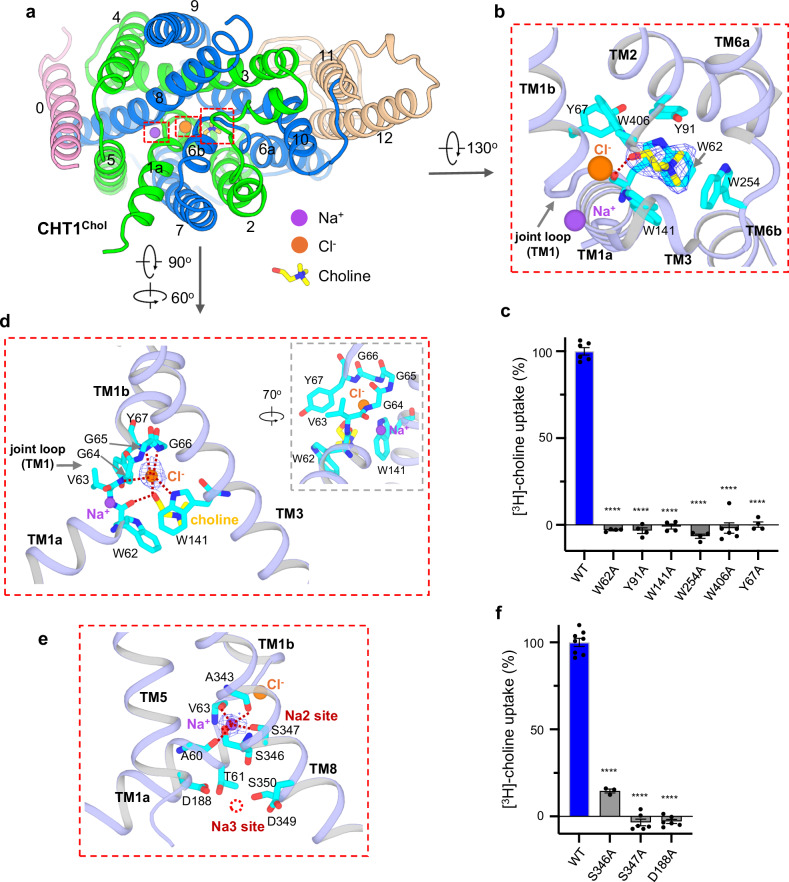
The substrate-bound CHT1^Chol^ structure. **a** Bottom view of the CHT1^Chol^ structure with the bound ions and choline highlighted in red dashed boxes. **b** Zoomed-in view of choline-binding in CHT1. The density (blue mesh) for choline is contoured at 6 σ. Key choline-interacting residues are shown in sticks. The bound Cl^–^ and Na^+^ are also shown for reference. **c** The effect of mutagenesis at the substrate-binding site on choline uptake. Data are mean ± SEM (*n* = 4–6 independent experiments) and are normalized against the measurement from the wild-type CHT1. One-way ANOVA; ****P ≤ 0.0001. **d** Zoomed-in view of Cl^–^ binding in CHT1. The density (blue mesh) for the Cl^–^ ion is contoured at 4 σ. Key Cl^–^-interacting residues are shown in sticks. The dotted lines mark the coordination between the Cl^–^ ion and the protein atoms. The inset provides an alternative view of the 63VGGGY67 region for enhanced clarity. **e** Zoomed-in view of Na^+^ binding at Na2 in CHT1. The density (blue mesh) for the Na^+^ ion is contoured at 4 σ. Key Na^+^ -interacting residues are shown in sticks. The dotted lines mark the coordination between the Na^+^ ion and the protein atoms. The surrounding residues for the Na3 site (red dotted circle) are also shown. **f** The effect of mutagenesis at Na2 and Na3 sites on choline uptake. Data are mean ± SEM (*n* = 3–8 independent experiments) and are normalized against the measurement from the wild-type CHT1. One-way ANOVA; ****P ≤ 0.0001.

The choline-bound CHT1 (CHT1^Chol^) structure was determined at 2.4 Å and the substrate can be unambiguously defined in the high-resolution EM map (Fig. 3a, b). Positioned at the center of the transporter, the substrate is surrounded by six aromatic residues (W62^TM1^, Y67^TM1^, Y91^TM2^, W141^TM3^, W254^TM6^, and W406^TM10^), among which the aromatic rings of W62, Y91, W141, and W254 cage the choline by forming cation–π interactions with its quaternary ammonium group. The bound choline is further stabilized by a hydrogen bond between its hydroxyl group and the backbone carbonyl of W62 (Fig. 3b; Supplementary Fig. S6a and Movie S1). All these choline-surrounding aromatic residues are highly conserved in CHT1 from invertebrates to mammals (Supplementary Fig. S4) and mutations of human CHT1 with these residues individually replaced by alanine all result in the loss of transport function without significantly affecting cell surface expression levels (Fig. 3c; Supplementary Fig. S7).

A well-defined density from a Cl^–^ ion is observed beside the bound choline (Fig. 3d; Supplementary Fig. S6a). The bound Cl^–^ is cuddled by the joint loop (63VGGGY67) between the two TM1 helices (TM1a and TM1b) and coordinated by the backbone nitrogen atoms from G64, G66, and Y67, the nitrogen atom from the indole sidechain of W141, and the hydroxyl group of choline, all within a distance ( ≤ 3.4 Å) optimal for Cl^–^ coordination (Fig. 3d; Supplementary Movie S1)^34,35^. The bound Cl^–^ ion plays three major roles in facilitating substrate binding: it directly interacts with the hydroxyl group of choline; it stabilizes the main chain conformation of the joint loop, allowing the backbone carbonyl of W62 to be positioned for a hydrogen-bonding interaction with the choline hydroxyl group; its interaction with W141 holds the indole ring of W141 in an orientation optimal for cation–π interaction with the quaternary ammonium group of choline. Thus, the Cl^–^ ion functions as a co-factor for choline-binding in CHT1, explaining the Cl^–^ dependence of the transporter activity.

A bound Na^+^ ion is also identified in CHT1^Chol^ at the Na2 site conserved in SGLT transporters (Fig. 3e; Supplementary Figs. S6a and S8a)^33,36^. It is located at the C-terminal end of TM1a close to the Cl^–^ ion and is chelated by the backbone carbonyls of A60, V63, and A343, and the sidechain hydroxyls of S346 and S347. Both S346A and S347A mutations lead to the loss of transport activity in CHT1 (Fig. 3f; Supplementary Fig. S7 and Movie S1), confirming the central role of this Na^+^ in co-transporting with the substrate as also seen in SGLTs. The Na^+^ coordination at Na2 by the backbone carbonyl of V63 also stabilizes the TM1’s joint loop and facilitates the neighboring Cl^–^ binding.

It is worth noting that some SGLT transporters and their bacterial homologs (such as sialic acid transporter SiaT) contain another Na^+^ site (Na3) right underneath Na2^33,37,38^. Those Na^+^-chelating residues at the Na3 site are conserved in CHT1, including D188, D349, and S350 (Fig. 3e; Supplementary Fig. S4), and the D188A mutation in CHT1 abolishes its transport activity (Fig. 3f; Supplementary Fig. S7), suggesting that Na3 is also present in CHT1 as the second Na^+^ site. However, no Na^+^ density is observed at Na3 in the inward-facing partially open CHT1^Chol^ structure because this site is fully exposed to the cytosolic solution with those ion-chelating residues slightly far apart for Na^+^ binding. As discussed later, the Na3 site is fully occluded from the cytosol in the outward-open structure with its surrounding residues closely positioned for Na^+^ binding (Supplementary Fig. S8a).

### Conformational changes between the inward-open apo and substrate-bound structures

**Fig. 4:**
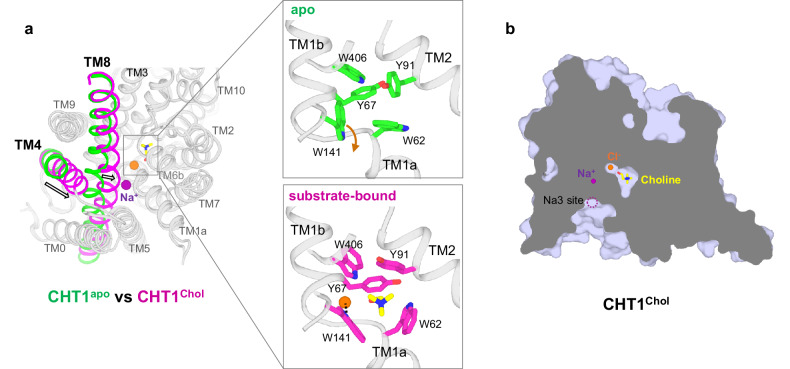
Conformational changes between the inward-open CHT1^apo^ and the substrate-bound CHT1^Chol^. **a** Structural comparison between CHT1^apo^ and CHT1^Chol^ viewed from the intracellular side. Only TM4 and TM8 are highlighted in color for clarity. Arrows mark the directions of the TM movements. The boxed insets provide zoomed-in views of the different sidechain positions of some Cl^–^ and substrate-interacting residues in these two structures. The orange arrow marks the flip of the W141 indole ring upon Cl^–^/choline-binding. **b** Side view of the cross-section of the surface-rendered CHT1^Chol^ illustrates its inward-facing partially open conformation. The bound Na^+^ (at Na2), Cl^–^, and choline are occluded from the intracellular solution. The dashed circle marks the solvent-exposed Na3 site.

Unlike the inward-open CHT1^apo^ structure, the substrate-bound CHT1^Chol^ adopts a partially open conformation. Two major conformational changes are observed from the apo to the substrate-bound state (Fig. 4a; Supplementary Fig. S6b and Movie S2). The first occurs at the substrate-binding site where the surrounding aromatic residues, including W62, Y67, Y91, W141, and W406, must reorient their sidechains to accommodate the substrate. Stabilized by the Cl^–^ ion, the W141 indole ring flips almost 180° and blocks the choline from the cytosol, forming a substrate release gate to the cytosol. The second conformational change occurs at TM8 whose middle part bulges toward the substrate site upon choline-binding. This movement is likely caused by Na^+^ binding at the Na2 site that bridges the interaction between the middle part of TM8 and TM1’s joint loop. Along with the bulging movement of TM8, the C-terminal part of TM4 also swings towards the substrate site because of the tight packing between TM4 and TM8 (Fig. 4a; Supplementary Fig. S6b). Consequently, the conformational changes at TM4 and TM8 shallow the intracellular vestibule and occlude the bound choline, Na^+^ (Na2 site), and Cl^–^ from the intracellular side, whereas the Na3 site remains exposed to the inside (Fig. 4b). Thus, the presence of an internal vestibule for the intracellular access of the Na3 site, albeit shallower than that in the inward-open apo state, defines the inward-facing partially open conformation of the CHT1^Chol^ structure.

### HC3-inhibited CHT1 in outward-open conformation

**Fig. 5:**
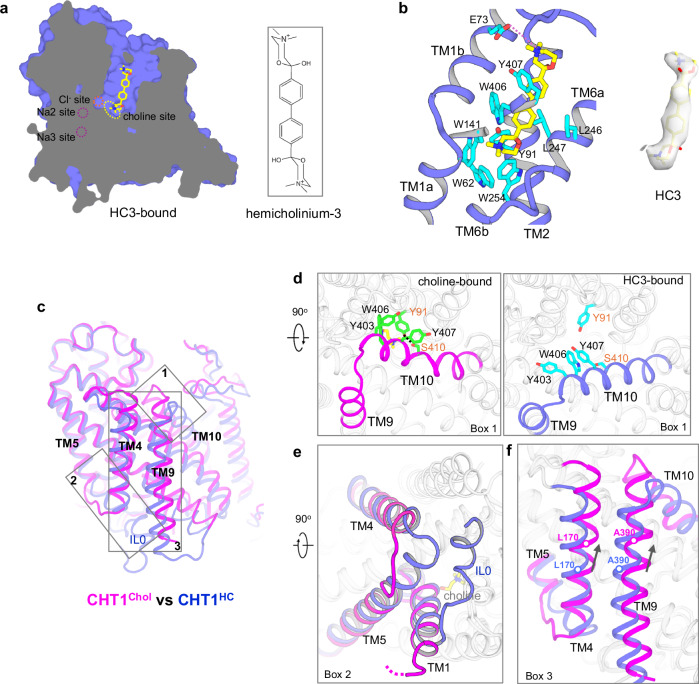
The structure of HC3-inhibited CHT1 in outward-open conformation. **a** Side view of the cross-section of the surface-rendered CHT1^HC^ with the bound HC3 (yellow sticks). The dotted circles and oval mark the locations of the ions and substrate-binding sites. **b** Zoomed-in view of the protein–inhibitor interactions and cryo-EM density of HC3 in the contour level of 1.5 in ChimeraX. **c** Structural comparison between CHT1^Chol^ and CHT1^HC^ in side view. TM0 is removed from both structures for clarity. The three areas where the major conformational changes occur are boxed. **d** Side-by-side view of the structural difference at the N-terminal region of TM10 (area in box 1) between CHT1^Chol^ (left) and CHT1^HC^ (right). **e** Zoomed-in view of the conformational changes at the N-terminal region of TM5 (area in box 2) between CHT1^Chol^ (magenta) and CHT1^HC^ (blue). **f** Zoomed-in view of the conformational changes at TM4 and TM9 (area in box 3) between CHT1^Chol^ (magenta) and CHT1^HC^ (blue). Arrows mark the upward movement of the helices from CHT1^HC^ to CHT1^Chol^. The hollow circles mark the C_α_ positions of L170 and A390 in the two structures and provide references for the one-turn helical movement between the two conformations.

The HC3-bound CHT1 structure was determined to be 3.7 Å and the transporter adopts an outward-open conformation (Fig. 5a). Although at a lower resolution, the EM density map for most of the transporter and the bound inhibitor is well-defined for modeling (Supplementary Fig. S3), particularly at the regions important for HC3 binding and substrate transport. HC3 is a symmetrical and elongated compound with a quaternary ammonium cation on each end (Fig. 5a inset). Similar to choline-binding, the quaternary ammonium on one end occupies the substrate-binding pocket in CHT1^HC^, stabilized by cation–π interactions with W62, W141, and W254 (Fig. 5b; Supplementary Fig. S9a, b). The rest of the rod-shaped inhibitor extends toward the extracellular solution with its quaternary ammonium on the opposite end stabilized by E73 and its two middle tandem benzene rings surrounded by hydrophobic or aromatic residues including Y91, L246, L247, W406, and Y407 (Fig. 5b). To verify the binding and inhibition of HC3, we performed mutagenesis on several of these HC3-interacting residues. L247A mutation slightly decreases the inhibition potency of HC3 whereas Y407A and S243R mutations significantly mitigate HC3 inhibition of choline uptake, supporting our structural observations (Supplementary Fig. S9c). The long stem of HC3 occupies the space of an otherwise solvent-accessible external vestibule and locks the transporter in an outward-open conformation.

### Conformational changes between the outward-open and substrate-bound structures

Comparison between CHT1^Chol^ and CHT1^HC^ reveals three major structural changes (Fig. 5c; Supplementary Movie S3). The first occurs at the N-terminal part of TM10 along with its short proceeding loop which swings away from the central substrate site in the CHT1^HC^ structure (Fig. 5d; Supplementary Fig. S9d). Several aromatic residues (Y403, W406, and Y407) at the N-terminus of TM10 that seal off the substrate from the extracellular side like a lid in CHT1^Chol^ flips away from the center in the CHT1^HC^ structure, generating a vestibule that exposes the substrate site to the extracellular solution. In CHT1^Chol^, the choline-interacting Y91 covers atop the substrate with a cation–π interaction and also stabilizes TM10 by forming a hydrogen bond with S410; however, these interactions are disrupted in CHT1^HC^ upon TM10 movement, causing the Y91 sidechain to slide away from the top of the substrate site. The structural difference at TM10 between CHT1^HC^ and CHT1^Chol^ likely represents the conformational change from open to close at the extracellular side of CHT1. As Y91 and W406 are key choline-interacting residues, substrate loading from the extracellular side and its ensuing interactions with these two residues would facilitate the closure of the external substrate pathway.

The second major structural change occurs at the N-terminal region of TM5 and its short proceeding loop (Fig. 5e; Supplementary Fig. S9d). The N-terminus of TM5 in CHT1^HC^ is about two helical turns longer than that in CHT1^Chol^ and also swings toward the center of the transporter. Furthermore, the internal loop 0 (IL0) between TM0 and TM1 which is disordered in the apo or substrate-bound structure forms a short helix in CHT1^HC^, making direct contact with the N-terminus of TM5 and plugging the entrance of the internal vestibule. Thus, the TM5 movement and the formation of a structured IL0 helix in CHT1^HC^ completely occlude the intracellular vestibule observed in the inward-open state. While the Na2 site remains the same in both structures, the TM5 movement in CHT1^HC^ also moves D188 closer to D349 and S350 on TM8, allowing these Na3 site residues to be optimally positioned for Na^+^ binding (Supplementary Fig. S8a). Although no Na^+^ density is observed at Na2 and Na3 sites in CHT1^HC^ likely caused by lower resolution, the geometries of both sites are almost identical to those observed in outward-open SiaT with bound Na^+^ ions (Supplementary Fig. S8a)^37^. To validate this, we performed molecular dynamics (MD) simulations of CHT1^HC^, which revealed stable Na^+^ occupancy at both Na2 and Na3 sites, consistent with our structural model (Supplementary Fig. S8b). When exposed to the high extracellular [Na^+^] environment, Na^+^ ions likely occupy both sites in the outward-open CHT1 and the Na^+^-bridged interaction between TM5 and TM8 at Na3 could stabilize the occlusion of the transporter from the intracellular side.

In essence, the above-discussed two major conformational changes manifest that the N-termini of TM5 and TM10 gate the substrate pathways from the intracellular and extracellular sides, respectively. The third major conformational change occurs at TM4 and TM9, two antiparallel helices that interlock these two gates (Fig. 5f; Supplementary Fig. S9d). Both TM4 and TM9 are connected to TM5 and TM10, respectively, via a well-structured short loop. From outward-open CHT1^HC^ to outward-closed CHT1^Chol^, the flip of TM10 N-terminus pulls TM9 together with TM4 upward by almost one helical turn (Fig. 5f). Through TM4, this upward movement exerts a dragging force to the N-terminus of TM5, unwinding its first two helical turns and thereby facilitating the inward opening of CHT1. Conversely, TM5-induced inward closing of CHT1 drives the downward movement of TM4 together with TM9, facilitating the flip of TM10 N-terminus and outward opening of the transporter. Thus, TMs 4 and 9 couple the inward and outward conformational changes, enabling the closing of the substrate site from one side to facilitate the opening from the other, and vice versa.

### ML352-inhibited CHT1 in inward-open conformation

**Fig. 6:**
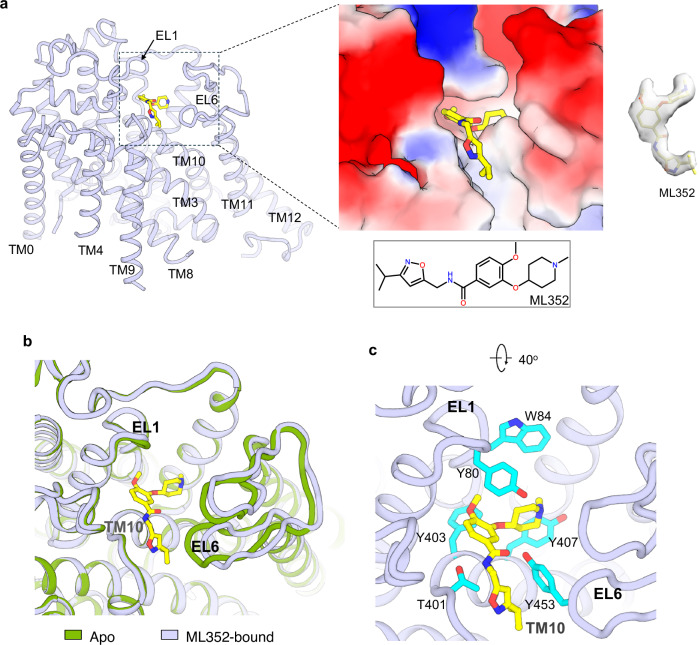
The structure of ML352-inhibited CHT1 in inward-open conformation. **a** The structure of CHT1^ML^ with the bound ML352 inhibitor on the external surface of the transporter. The zoomed-in view of the surface-rendered CHT1^ML^ illustrates the space-filling binding of ML352. Cryo-EM density of ML352 in the contour level of 0.42 in ChimeraX. **b** Structural comparison between CHT1^apo^ and CHT1^ML^ illustrates subtle structural change at the EL6 loop between the two. **c** Zoomed-in view of the protein–inhibitor interactions.

The structure of CHT1 in complex with ML352 was determined to be 2.9 Å resolution and the transporter is virtually identical to the inward-open apo structure except for a slight displacement of the EL6 loop between TM11 and TM12 (Fig. 6a, b). Unlike HC3, ML352 does not compete for the substrate pocket but rather binds on the external surface of the transporter (Fig. 6a). Sitting atop the N-terminal region of TM10, the shape of the inhibitor fits quite well into a surface groove formed by predominantly aromatic residues from TM10 (T401, Y403, and Y407), the EL1 loop between TM1 and TM2 (Y80 and W84), and the EL6 loop between TM11 and TM12 (Y453) (Fig. 6c; Supplementary Fig. S10a). Interestingly, mutations of the hydrophobic residues surrounding ML352 (Y80A, Y453A, and Y407A) resulted in similar inhibition profiles to the wild-type, indicating that these substitutions have minor effects on ML352 binding (Supplementary Fig. S10b). As ML352 engages in extensive hydrophobic interactions with CHT1 in the groove over a large surface area of the protein, we reason that a single alanine mutation of these aromatic residues may not be sufficient to abolish its binding. To further investigate this, we performed MD simulations of CHT1 in the ML352-bound state, which confirmed that ML352 remains stably bound within the pocket as observed in our structural model (Supplementary Fig. S10c). This spacing-filling occupation of the inhibitor glues the N-terminal region of TM10 to the EL1 and EL6 loops, preventing it from undergoing conformational change to open the extracellular vestibule. Thus, ML352 inhibits CHT1 by trapping the transporter in a closed conformation on its extracellular side, which in turn stabilizes the transporter in an inward-open state.

## Discussion

Our high-resolution structures of CHT1 combined with the functional analysis reveal the structural mechanisms underlying substrate and ion binding, substrate transport, and drug inhibition of CHT1 symporter. The transport function of CHT1 requires both Na^+^ and Cl^–^ ions. Three ion-binding sites, two for Na^+^ and one for Cl^–^, are unambiguously defined in the structures. Both ions are indispensable cofactors for high-affinity choline-binding and are likely transported together with the substrate in a 2:1:1 stoichiometry. Thus, CHT1 utilizes the chemical gradients from both Na^+^ and Cl^–^ to facilitate the uptake of the positively charged choline molecules.

The structures of CHT1 in complex with HC3 and ML352 reveal two distinct inhibitory mechanisms by these two compounds. The rod-shaped HC3 competitively binds at the substrate site using its quaternary ammonium on one end while its long stem locks the transporter in an outward-open conformation by occupying the external vestibule space. ML352, on the other hand, does not compete for the substrate site. It binds at the external surface of CHT1 and traps the transporter in an inward-open conformation by stabilizing TM10 in the closed conformation on the extracellular side.

**Fig. 7:**
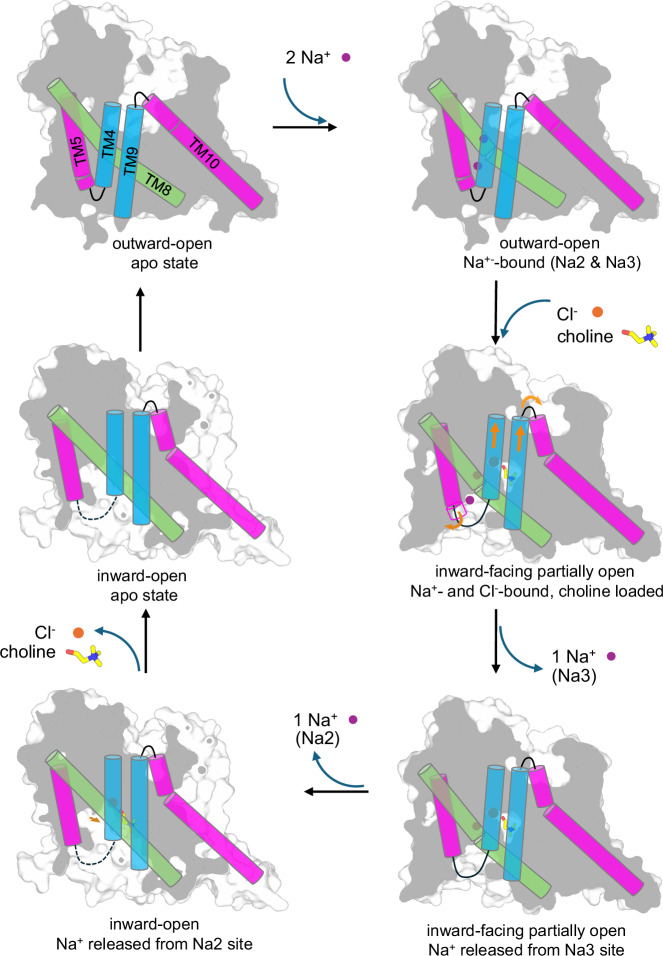
A working model for the Na^+^ and Cl^–^-dependent choline transport in CHT1. The orange arrows mark the conformational changes driven by the choline-binding from the extracellular side.

The CHT1 structures in multiple conformations also allow us to propose a plausible working model for the CHT1-mediated choline translocation process across the cell membrane (Fig. 7; Supplementary Movie S4). Starting with the outward-open conformation in which the substrate and ion binding sites are exposed to the extracellular solution with high [Na^+^] and [Cl^–^], the choline translocation is initiated by Na^+^ occupation at Na2 and Na3 sites. Na^+^ binding at Na3 stabilizes the outward-open state by bridging the interaction between TM5 gate and TM8 whereas Na^+^ binding at Na2 stabilizes the joint loop in the middle of TM1 and facilitates Cl^–^ binding. Subsequently, Cl^–^ binds and interacts with key residues at the substrate site to facilitate the choline upload. Upon occupying the substrate site, choline engages in the protein–ligand interactions with Y91 and W406 and drives the conformational change at TM10 to close the extracellular pathway. Mediated by the upward movement of TMs 4 and 9, the gate-closing conformational change at TM10 is coupled to the N-terminus of TM5, causing the unwinding of its first two helical turns and the disordering of the adjacent IL0 helix. By contrast, IL0 adopts a structured helix in the outward-facing state, acting as a gatekeeper for the intracellular vestibule^39^. This structural change partially opens the ions/substrate-loaded CHT1 to the intracellular solution with low [Na^+^] and [Cl^–^] and exposes the Na3 site for the first Na^+^ release to generate the inward-facing partially open state. The subsequent second Na^+^ release from Na2 ensues the straightening movement of TM8, rendering the transporter into an inward-open state ready for Cl^–^ and choline release. We speculate that the Cl^–^ ion is released first, which in turn weakens the choline-binding and also removes the constraint on the W141 sidechain, allowing its indole ring to flip for choline release to the cytosol.

CHT1 dysfunction is implicated in several human diseases, including distal hereditary motor neuropathy and congenital myasthenic syndrome^40^. We map these disease-associated mutants onto the CHT1 structure to shed light on the molecular mechanisms underlying these disorders (Supplementary Fig. S11). Clinically identified CHT1 mutations exhibit diverse pathogenic mechanisms. Frameshift mutations commonly truncate the C-terminus, a region crucial for protein trafficking to the cell surface (Supplementary Fig. S11a), resulting in reduced protein levels in the plasma membrane and thereby impairing choline uptake^19,20^. Missense mutations within transmembrane domains frequently disrupt critical residues involved in substrate binding or ion coordination. For instance, G65, a key residue in the joint loop (63VGGGY67) connecting the two TM1 helices, is essential for Cl^–^ binding. The G65E mutation can potentially abolish Cl^–^ binding important for substrate uptake (Supplementary Fig. S11b)^23^. Similarly, D349 directly coordinates Na^+^ at the Na3 site, facilitating choline transport (Supplementary Fig. S11b). The D349N mutation would weaken or disrupt this interaction, leading to a complete loss of transport function^22^. Additionally, many patient-derived mutations occur on the protein surface, including in the intracellular and extracellular loops^23,24,40^. While not directly involved in substrate transport, these mutations likely contribute to choline uptake deficiency by affecting protein folding, stability, or cellular trafficking.

## Materials and methods

### Cell lines

*Spodoptera frugiperda* Sf9 cells were used to generate baculovirus and maintained in Sf-900 II SFM medium at 27 °C with shaking. HEK293S GnTI- cells were maintained in FreeStyle 293 Expression Medium at 37 °C, 8% CO_2_, 130 rpm in a Reach-In CO_2_ Incubator (ThermoFisher Scientific) throughout. HEK293 cells used for uptake and inhibition assays were grown in DMEM high glucose media supplemented with 10% fetal bovine serum (FBS) and kept in a 37 °C, 5% CO_2_ incubator. HEK293 and HEK293S GnTI- cell lines were purchased from and authenticated by the American Type Culture Collection (ATCC).

### Protein expression and purification

Full-length human CHT1 (NCBI accession: NP_068587.1) containing a C-terminal Strep-tag was cloned into a pEZT-BM vector^41^ and expressed in HEK293S GnTI- cells (ATCC) using the BacMam system^42^. Bacmids were synthesized using *Escherichia coli* DH10Bac cells (ThermoFisher Scientific), and the baculoviruses were produced in Sf9 cells using Cellfectin II reagent (ThermoFisher Scientific). For protein expression, cultured GnTI- cells were infected with the baculoviruses at a ratio of 1:20 (virus:GnTI-, v/v) for 10 h. 10 mM sodium butyrate was then introduced to boost protein expression level, and cells were cultured in suspension at 30 °C for another 60 h before being harvested by centrifugation at 4000× *g* for 15 min. All purification procedures were carried out at 4 °C. The cell pellet was resuspended in lysis buffer (25 mM HEPES pH 7.4, 300 mM NaCl, 2 μg/mL DNase I, 0.5 μg/mL pepstatin, 2 μg/mL leupeptin, 1 μg/mL aprotinin, and 0.1 mM PMSF) and homogenized by sonication. CHT1 was extracted with 2% (w/v) N-dodecyl-β-D-maltopyranoside (DDM, Anatrace) supplemented with 0.2% (w/v) cholesteryl hemisuccinate (CHS, Sigma-Aldrich) by gentle agitation for 2 h. After extraction, the supernatant was collected by centrifugation at 40,000× *g* for 30 min and incubated with Strep-Tactin affinity resin (IBA) for 1 h. The resin was then collected on a disposable gravity column (Bio-Rad) and sequentially washed with 30 column volumes of buffer A (25 mM HEPES pH 7.4, 150 mM NaCl) supplemented with 0.03% (w/v) lauryl maltose neopentyl glycol (LMNG, Anatrace). CHT1 was eluted in buffer A supplemented with 0.004% LMNG and 50 mM biotin. The protein eluate was concentrated and further purified by size-exclusion chromatography on a Superdex200 10/300 GL column (GE Healthcare) in buffer A with 0.004% LMNG. The peak fractions were collected and concentrated to about 3 mg/mL for cryo-EM analysis. To prepare the protein samples in complex with substrate and inhibitors, 25 mM choline chloride, 1 mM HC3, or 0.5 mM ML352 was added to the protein samples about 2 h before EM grids preparation for data collection.

### Cryo-EM sample preparation and data acquisition

Human CHT1 samples ( ~3 mg/mL) in various conditions were applied to a glow-discharged Quantifoil R1.2/1.3 300-mesh gold holey carbon grid (Quantifoil, Micro Tools GmbH, Germany), blotted for 4.0 s under 100% humidity at 4 °C and plunged into liquid ethane using a Mark IV Vitrobot (FEI). For HC3-bound CHT1, raw movies were acquired on a Titan Krios microscope (FEI) operated at 300 kV with a K3 camera (Gatan) at 0.84 Å per pixel and a nominal defocus range of L –1.0 to –2.0 μm. Each movie was recorded for about 5 s in 60 subframes with a total dose of 60 e^–^/Å^2^. For other samples, raw movies were acquired on a Titan Krios microscope operated at 300 kV with a Falcon 4i (ThermoFisher Scientific) at 0.738 Å per pixel and a nominal defocus range of –0.8 to –1.8 μm. Each movie was recorded for 4 s with a total dose of 60 e^–^/Å^2^.

### Image processing

Cryo-EM data were processed following the general scheme described below with some modifications to different datasets (Supplementary Fig. S2). First, movie frames were motion-corrected and dose-weighted using MotionCor2^43^. The CTF parameters of the micrographs were estimated using the GCTF program^44^. After CTF estimation, micrographs were manually inspected to remove images with bad defocus values and ice contamination. Particles were picked using the program crYOLO and extracted with a binning factor of 3 in RELION^45,46^. Extracted particles were subjected to 2D classification. Particles from some good 2D classes were selected for generating an ab initio model for the following 3D classification. The particles from the best-resolving 3D class were then re-extracted with the original pixel size and subjected to heterogenous 3D refinement, non-uniform refinement, CTF refinement, local 3D refinement, and Bayesian polishing. All resolution was reported according to the gold-standard Fourier shell correlation using the 0.143 criterion. Local resolution was estimated using cryoSPARC^47^.

### Model building, refinement, and validation

The EM maps of human CHT1 show high-quality density for de novo model building in Coot, facilitated by the predicted structure from Alphafold^48,49^. Models were manually adjusted in Coot and refined iteratively against maps using Phenix^50^. The final CHT1 structural model contains residues 2–30 and 37–518. The C-terminal residues 519–580 of CHT1 are disordered in the structure. The statistics of the geometries of the models were generated using Phenix and MolProbity^50,51^. All the figures were prepared in PyMol (Schrödinger), UCSF Chimera, or UCSF ChimeraX^52,53^.

### [^3^H]-Choline uptake assay

The radioactive choline uptake assay was performed following a published protocol^32^. Wild-type human CHT1 and its mutants were cloned into the pEZT-BM vector. HEK293 cells were maintained in Dulbecco’s Modified Eagle Medium (DMEM) high glucose medium supplemented with 10% FBS and 1× penicillin-streptomycin solution at 37°C and 5% CO_2_. HEK293 cells were seeded onto poly-D-lysine-coated 24-well plates at a density of 0.15 × 10^6^ cells per well and cultured at 37 °C for 12 h. The cells were then transfected with the plasmids containing wild-type CHT1 or mutants using FuGene HD following the manufacturer’s protocol and cultured for another 48 h. Subsequently, the cell culture medium was removed by aspiration, and the cells were washed twice with Krebs-Ringer’s-HEPES (KRH) buffer (130 mM NaCl, 1.3 mM KCl, 1.2 mM MgCl_2_, 2.2 mM CaCl_2_, 1.2 mM KH_2_PO4, 10 mM glucose and 20 mM HEPES pH 7.4) and pre-incubated in KRH buffer for 1–2 h at 37 °C before being used for the uptake assay. Radioactive choline uptake was initiated by replacing the KRH buffer in the cell culture well with the reaction solutions containing KRH buffer with various concentrations of choline supplemented with 10% (for most experiments) or 1% (for the time-dependent uptake measurement and the concentration-dependent kinetic assay) of [^3^H]-choline (American Radiolabeled Chemicals) at room temperature. Uptake was terminated by removing the reaction solutions at different time points followed by washing the cells three times with ice-cold KRH buffer. The cells were then solubilized in 1% SDS, 0.2 M NaOH, and radioactivity was measured using a Beckman Coulter LS6500 liquid scintillation counter.

Except for the time-dependent assay shown in Supplementary Fig. S1a, all other choline uptake assays were terminated at the 10-min time point. For most experiments, the reaction solutions contained 0.1 μM of total choline with 10% of [^3^H]-choline. Due to higher concentrations of choline used in the time-dependent uptake measurement and the substrate concentration-dependent kinetic assay, the choline in the reaction solutions for those experiments contained 1% of [^3^H]-choline. HEK293 cells transfected with the empty pEZT-BM vector were used as the control for background radioactivity measurement subtracted in all quantitative data analyses. Transport activities were normalized based on cell surface expression levels. For inhibition assay, HC3 and ML352 were added to the pre-incubation buffer at various concentrations and were also included in the reaction solutions. In Na^+^- and Cl^–^-dependent uptake assays, the 130 mM NaCl in the reaction solution was replaced by sodium methanesulfonate (NaMS), KCl, or N-methyl-d-glucamine chloride (NMDG-Cl) at an equal concentration. Data for choline concentration-dependent update were fitted with the Michaelis–Menten equation. Data for concentration-dependent HC3 and ML352 inhibition were fitted with three-parameter dose–response curves. Statistical analyses of data were performed using GraphPad Prism 9. Quantification methods and details are described in each relevant section of the methods or figure legends.

### Cell surface biotinylation assay

Biotinylation assays were performed to determine the expression levels of wild-type and mutant CHT1 on the cell surface. HEK293 cells were transfected with pEZT-BM plasmids encoding wild-type CHT1 or its mutants with a C-terminal Strep-tag using FuGene 6 reagent. After being washed three times with ice-cold PBS, the cells were suspended at a concentration of 15 × 10^6^ cells/mL in PBS (pH 8.0), followed by the addition of 16 μL of freshly prepared 10 mM Sulfo-NHS-SS-Biotin (ThermoFisher Scientific, cat# 21331). After incubation at room temperature for 30 min, cells were washed three times with ice-cold TBS (20 mM Tris-HCl, pH 7.5, 150 mM NaCl) to quench and remove the non-reacted biotinylation reagent. The cells were lysed in 150 μL of TBS supplemented with 1% (w/v) DDM, 0.1% (w/v) CHS, and 1× protease inhibitor cocktail (ApexBio, cat# K1010). Each sample of cell lysates was centrifuged at 20,000× *g* and incubated with 30 μL of 50% (v/v) NeutrAvidin agarose (ThermoFisher Scientific, cat# 29200) by rotation at 4 °C for 30 min. The agarose beads were then washed three times with buffer W (TBS supplemented with 0.1% DDM and 0.01% CHS). Biotinylated proteins bound to the beads were eluted by incubation with buffer W containing 20 mM TCEP-HCl (MilliporeSigma, cat# 646547) at room temperature for 10 min. The eluted proteins were separated on SDS-PAGE gels and transferred to nitrocellulose membranes for immunoblot analysis. After blocking, primary antibody incubation, and secondary antibody incubation, the membranes were developed for 2 min at room temperature using SuperSignal West Pico PLUS Chemiluminescent Substrate (ThermoFisher Scientific, cat# 34580) and then imaged using the LI-COR Odyssey Fc imaging system.

The following primary antibodies were used for probing corresponding proteins: anti-Strep-tag mouse monoclonal antibody (IBA Lifesciences, cat# 2-1507-001, 1:10,000 dilution) for wild-type CHT1 and mutants; anti-Na^+^/K^+^-ATPase subunit alpha-1 rabbit monoclonal antibody (Abcam, cat# ab76020, 1:10,000) for plasma membrane marker; anti-beta Actin mouse monoclonal antibody (Santa Cruz, cat# sc-69879, 1:2000 dilution) for cytosolic marker. Horseradish peroxidase-conjugated goat anti-rabbit or anti-mouse IgG (H + L) secondary antibody (Jackson ImmunoResearch, cat# 111-035-144 or 115-035-146, 1:10,000 dilution) was used for chemiluminescence detection.

### MD simulations

Atomistic models of CHT1 in apo, choline-bound, HC3-bound, and ML352-bound states were generated based on corresponding cryo-EM structures. Missing regions within TM helices were modeled as unstructured loops using AlphaFold2 predictions as a template. These models were embedded in a POPC lipid bilayer, and solvated in a 0.15 M NaCl solution. The dimensions of the simulation box were 8.5 nm × 8.5 nm × 8.2 nm in the *x*, *y*, and *z* directions, respectively, totaling ~55,000 atoms. Protein-lipid orientation was optimized using the OPM server. System setup and energy minimization were performed using CHARMM-GUI^54^, followed by six-step equilibration. Production MD simulations were conducted under NPT conditions using the CHARMM36m force field for proteins and lipids^55^, and the TIP3P water model. The force field parameters for choline, HC3, and ML352 were obtained from CHARMM general force field (CGenFF)^56^. Residue protonation states were determined based on PROPKA3.1 predictions^57^ and local environment analysis, resulting in the protonation of Glu138. During the MD simulations, the temperature was maintained at 310 K with a Nosé–Hoover thermostat and a coupling constant of 1 ps, and pressure was kept at 1.0 bar using the Parrinello–Rahman barostat and a time coupling constant of 5 ps. A switch function with a starting distance of 1.0 nm was employed for a van der Waals cut-off of 1.2 nm. Short-range electrostatic interactions were also truncated at 1.2 nm, while long-range electrostatic interactions were computed via the particle mesh Ewald decomposition algorithm with a 0.12-nm mesh spacing. Three independent 1500 ns MD simulations were performed for the apo, choline-bound, and HC3-bound states of CHT1, with one simulation applying backbone position restraints on CHT1 to maintain the cryo-EM structure. The ML352-bound state was subjected to three independent 1000 ns unrestrained MD simulations. All simulations were conducted using GPU-accelerated Gromacs software (versions 2022.5 or 2024.2)^58^. Trajectory analysis was conducted with Gromacs gmx tools.

## Supplementary information

SUPPLEMENTAL MATERIAL

Movie S1, Local structures at the ion- and substrate-binding sites of CHT1^Chol^

Movie S2, Conformational changes between CHT1^apo^ and CHT1^Chol^

Movie S3, Conformational changes between CHT1^HC^ and CHT1^Chol^

Movie S4, Working model for the Na^+^- and Cl^-^-dependent choline transport in CHT1

## Data Availability

The cryo-EM density maps of the human CHT1 have been deposited in the Electron Microscopy Data Bank (EMDB) under accession numbers EMD-44497 for apo state, EMD-44498 for choline-bound state, EMD-44593 for HC3-bound state, and EMD-44499 for ML352-bound state. Atomic coordinates have been deposited in the Protein Data Bank (PDB) under accession numbers 9BFI for the apo state, 9BFJ for the choline-bound state, 9BIM for the HC3-bound state, and 9BFK for the ML352-bound state.
