# Supplementary material for: Structural mechanisms of human sodium-coupled high-affinity choline transporter CHT1
Source: Cell Discov. 2024 Nov 26;10:116. doi: 10.1038/s41421-024-00731-7 (PMC11589582; doi:10.1038/s41421-024-00731-7)
Supplement: Supplementary file 1 — SUPPLEMENTAL MATERIAL [file 41421_2024_731_MOESM1_ESM.pdf]

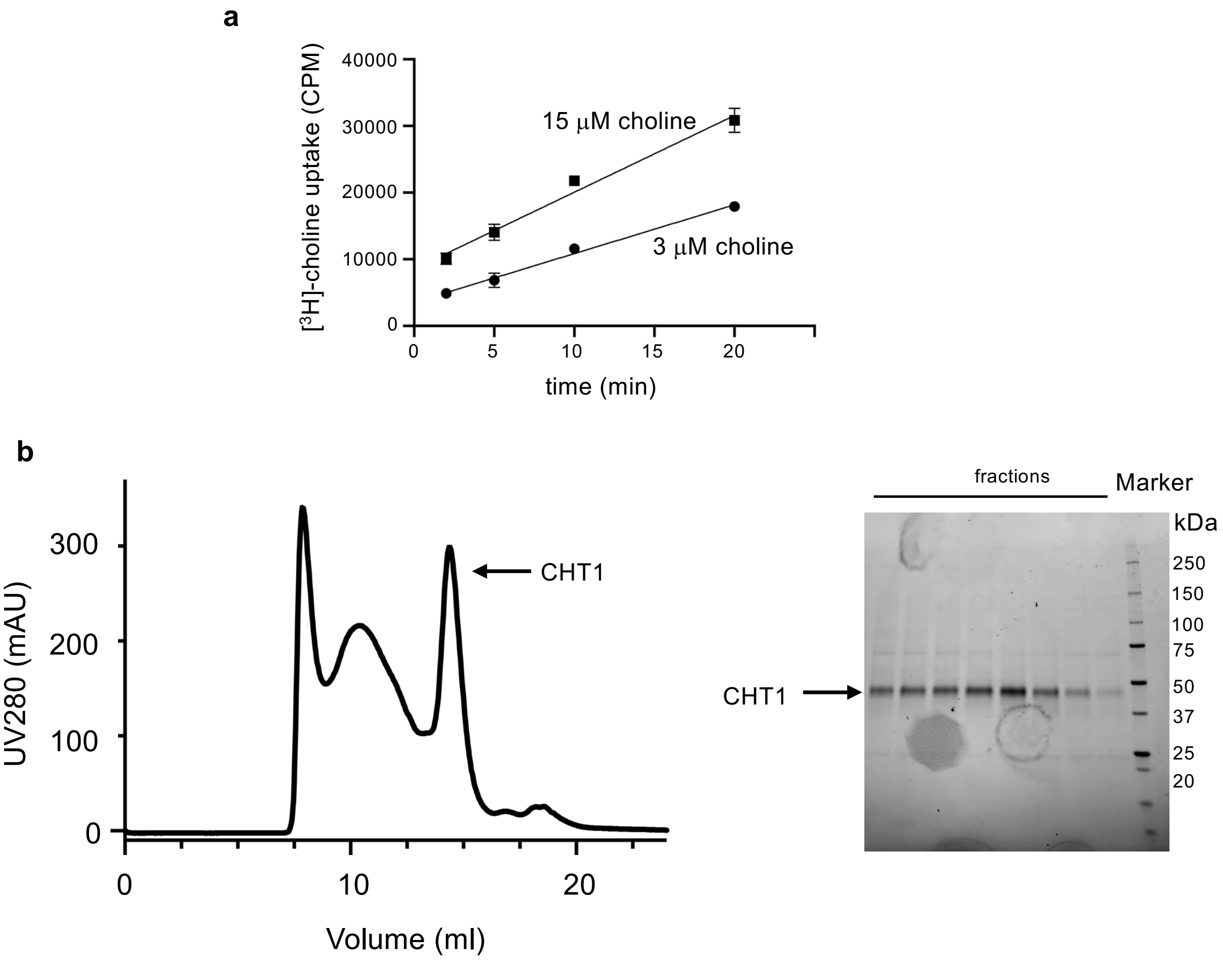


**Fig. S1 Biochemical characterization of CHT1.**
**a,** Time-dependent accumulation of radioactivity in CHT1-expressing HEK293 cells. 3 or 15 μM of choline was added to the extracellular solution, containing 1% radioactive [$^3$H]-choline. Data are mean ± SEM (n = 3 independent experiments).
**b,** Representative size exclusion chromatography profile and SDS-PAGE analysis of purified CHT1 in LMNG.

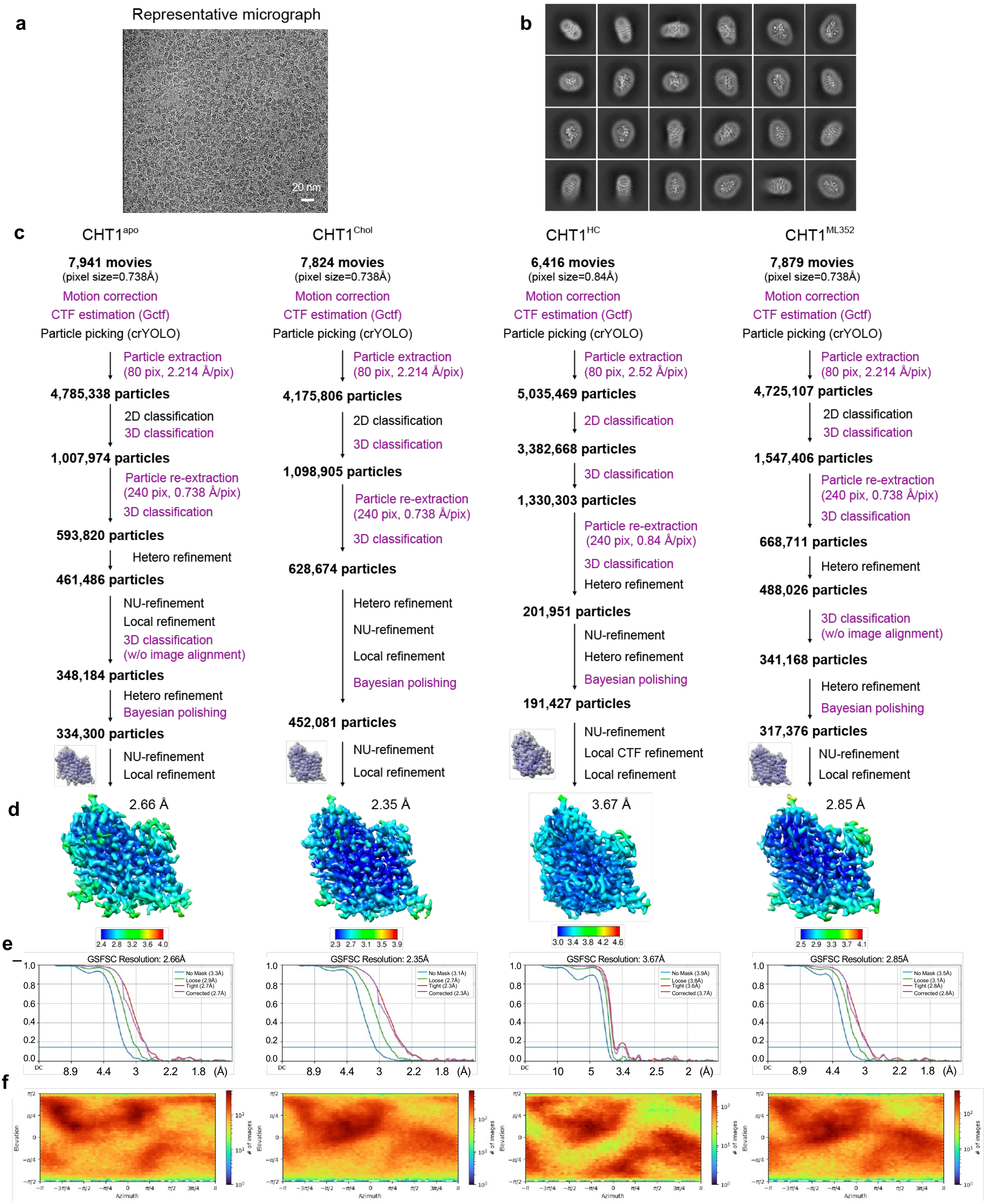


**Fig. S2 Cryo-EM data processing scheme.**

**a,** A representative micrograph. Scale bar is at 20 nm. **b,** Representative 2D class averages. **c,** Flow chart of the cryo-EM data processing procedure. **d,** Final maps colored by local resolution. **e,** Fourier Shell Correlation curves showing the overall resolution. **f,** Plots of viewing direction distribution calculated in cryoSPARC.

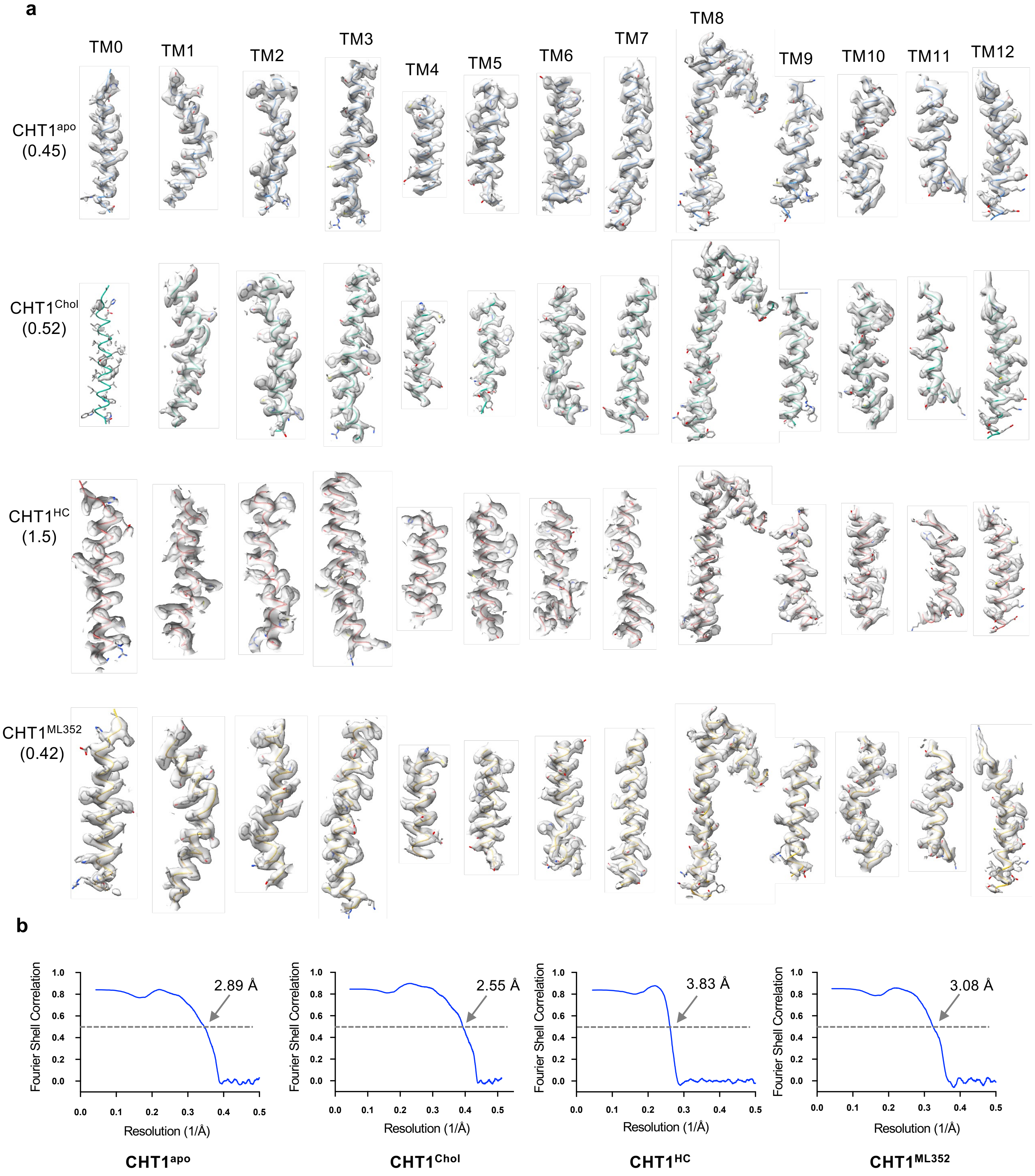


**Fig. S3 Maps and refined models of the CHT1 structures.**

**a,** Density maps are contoured at the indicated threshold level (numbers in brackets) using the ChimeraX software.

**b,** FSC curves calculated between the cryo-EM map and structural model.

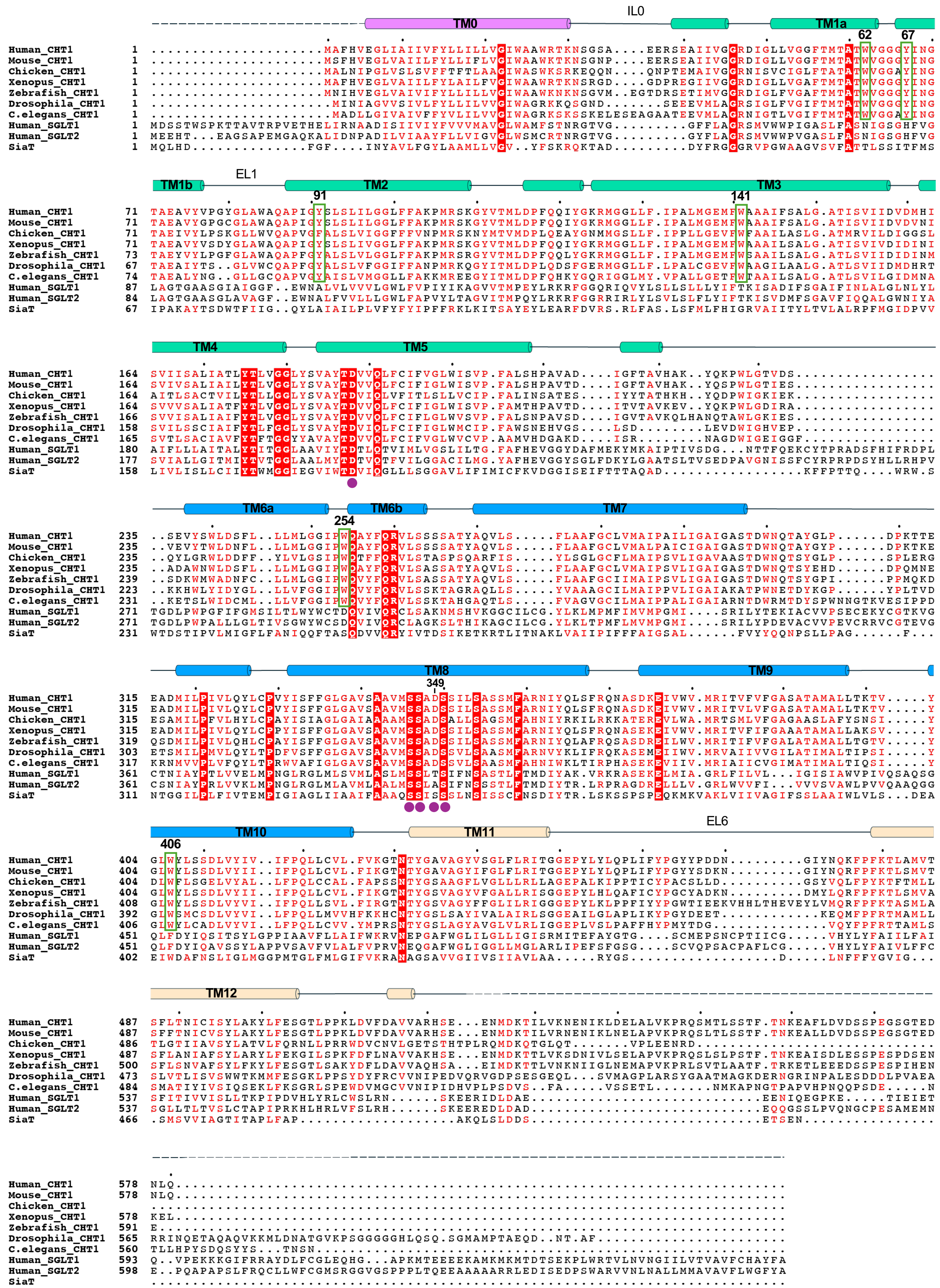


**Fig. S4 Sequence alignment of CHT1s from various species, human SGLT1&2, and *Proteus mirabilis* sialic acid transporter SiaT**. Green boxes enclose the choline-interacting residues. Purple spheres mark the residues whose sidechains participate in the $Na^+$ binding at Na2 and Na3 sites.

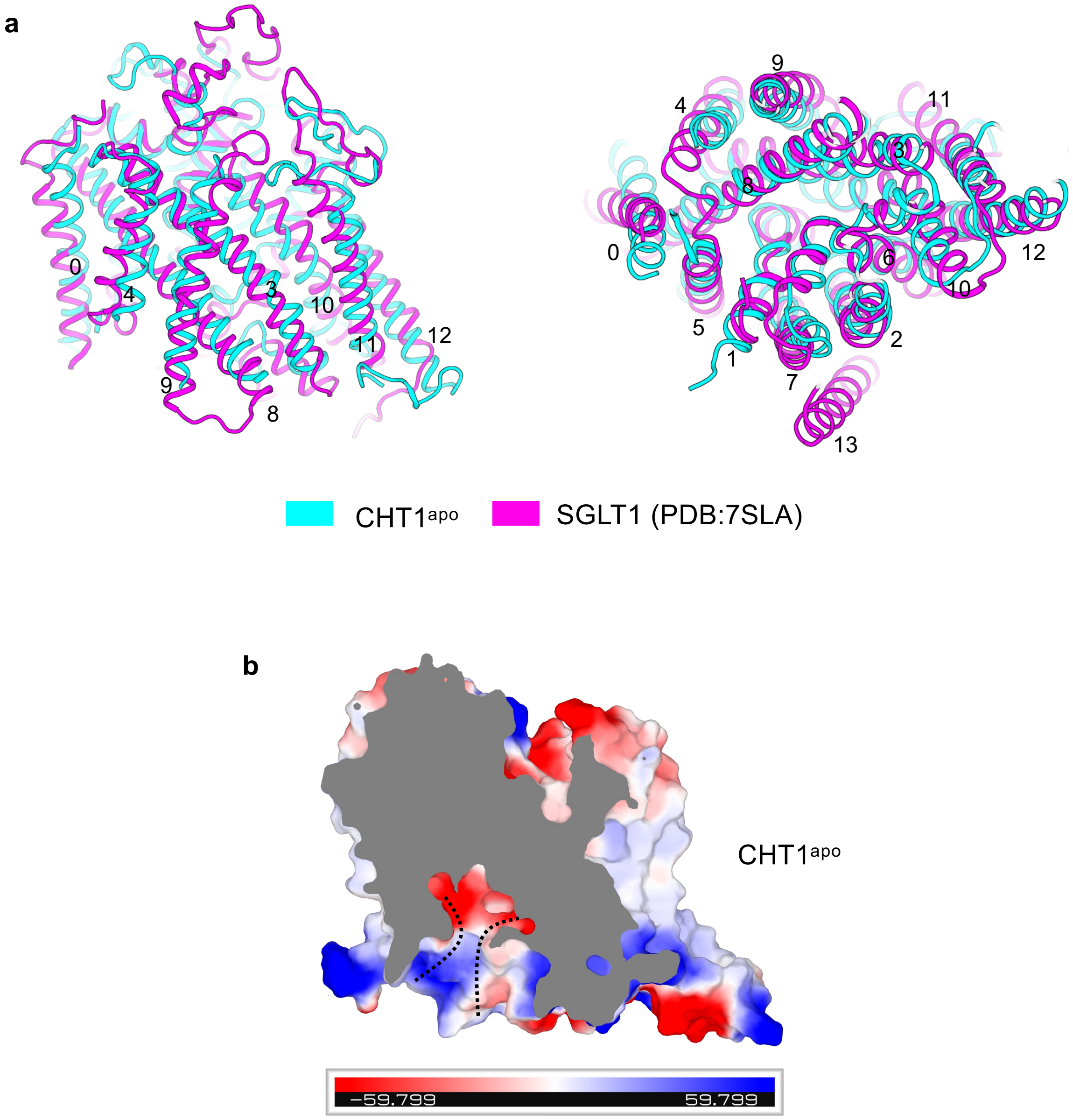


**Fig. S5 Structure of CHT1$^{apo}$ and its comparison with human SGLT1.**

**a,** Superimposition of CHT1$^{apo}$ and human SGLT1.

**b,** Electrostatic potential map of the inward-open CHT1$^{apo}$ structure, highlighting an open hydrophilic intracellular vestibule.

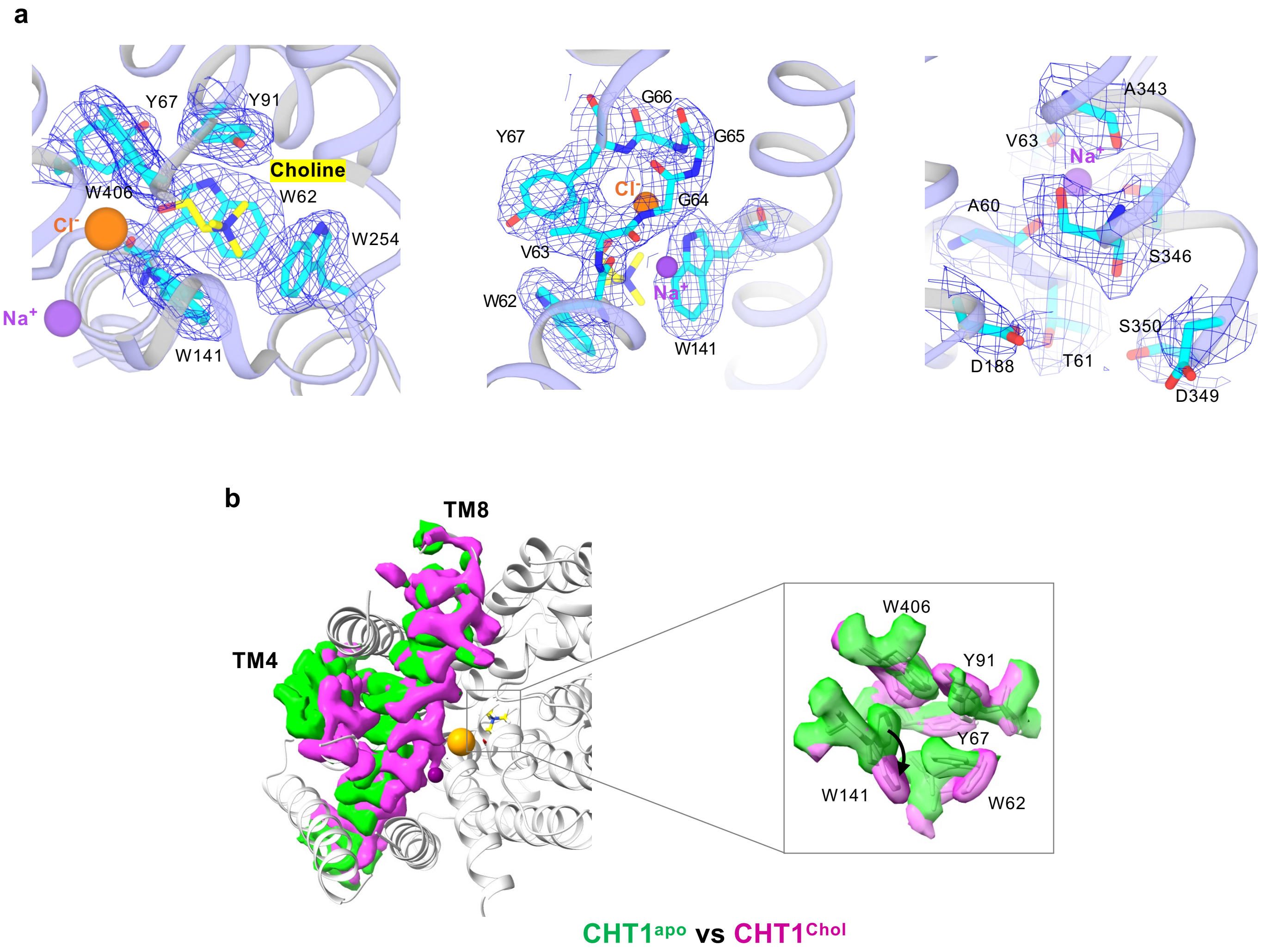


**Fig. S6 Cryo-EM density maps of CHT1$^{Chol}$.**

**a,** Cryo-EM densities of the residues coordinating choline, $Na^{+}$ and $Cl^{-}$, as shown in Fig. 3b, d, and e. The densities (blue mesh) are contoured at 6 σ, 6 σ and 4 σ, respectively.

**b,** Density comparison between CHT1$^{apo}$ and CHT1$^{Chol}$ at TM4, TM8 and the choline coordinating residues.

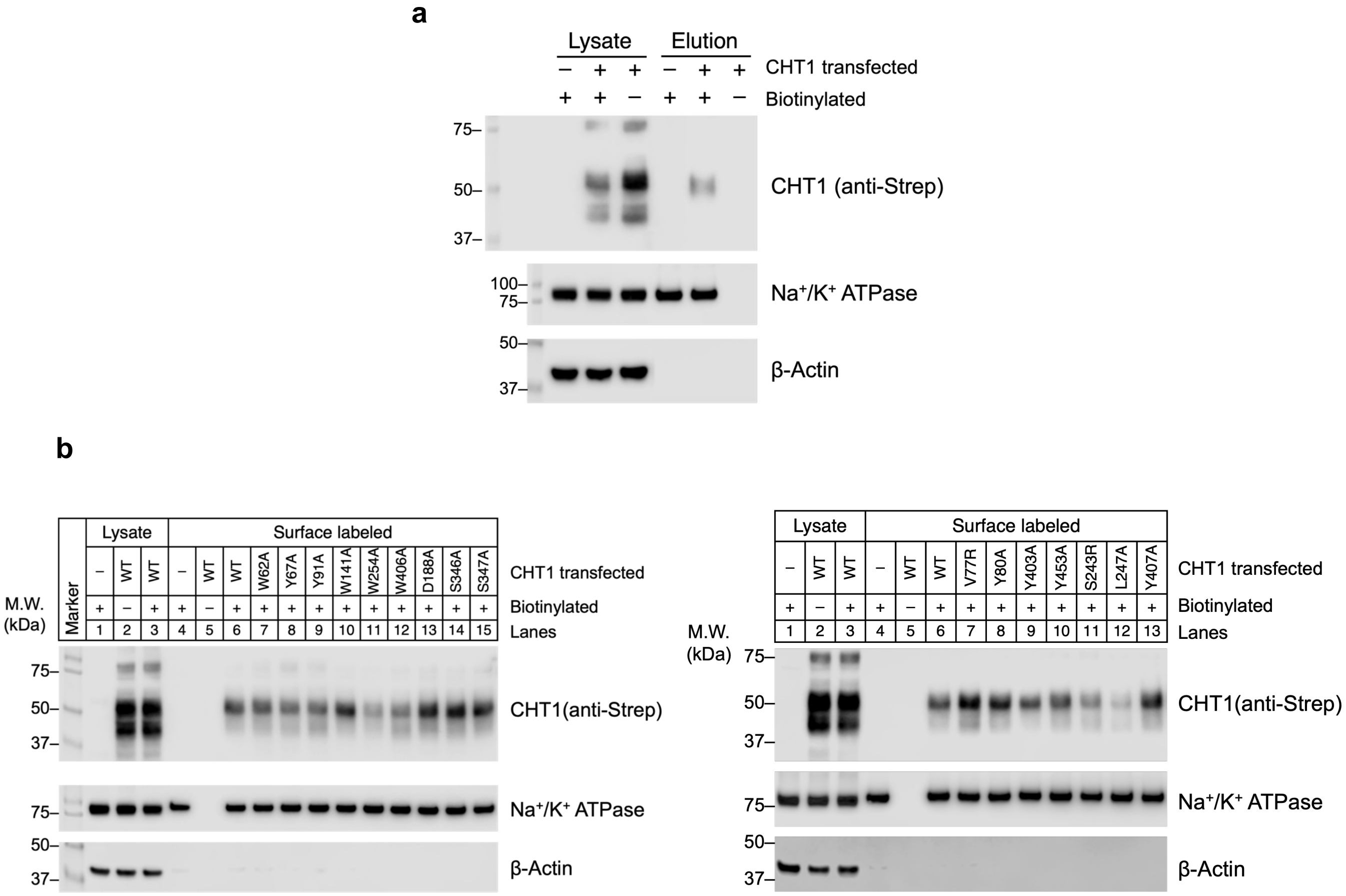


**Fig. S7 Cell surface expression of CHT1 and its mutans.**

**a,** Cell surface proteins were biotinylated and pulled down using NeutrAvidin agarose beads. The successful biotinylation of the plasma membrane marker $Na^{+}/K^{+}$-ATPase, and the lack of biotinylation of the cytosolic marker Actin, confirm that CHT1 biotinylation reflects its localization on the plasma membrane.

**b,** Expression levels of CHT1 and its mutants on the plasma membrane, as determined by biotinylation and pull-down assays. Transport activities were normalized based on these measured cell surface expression levels.

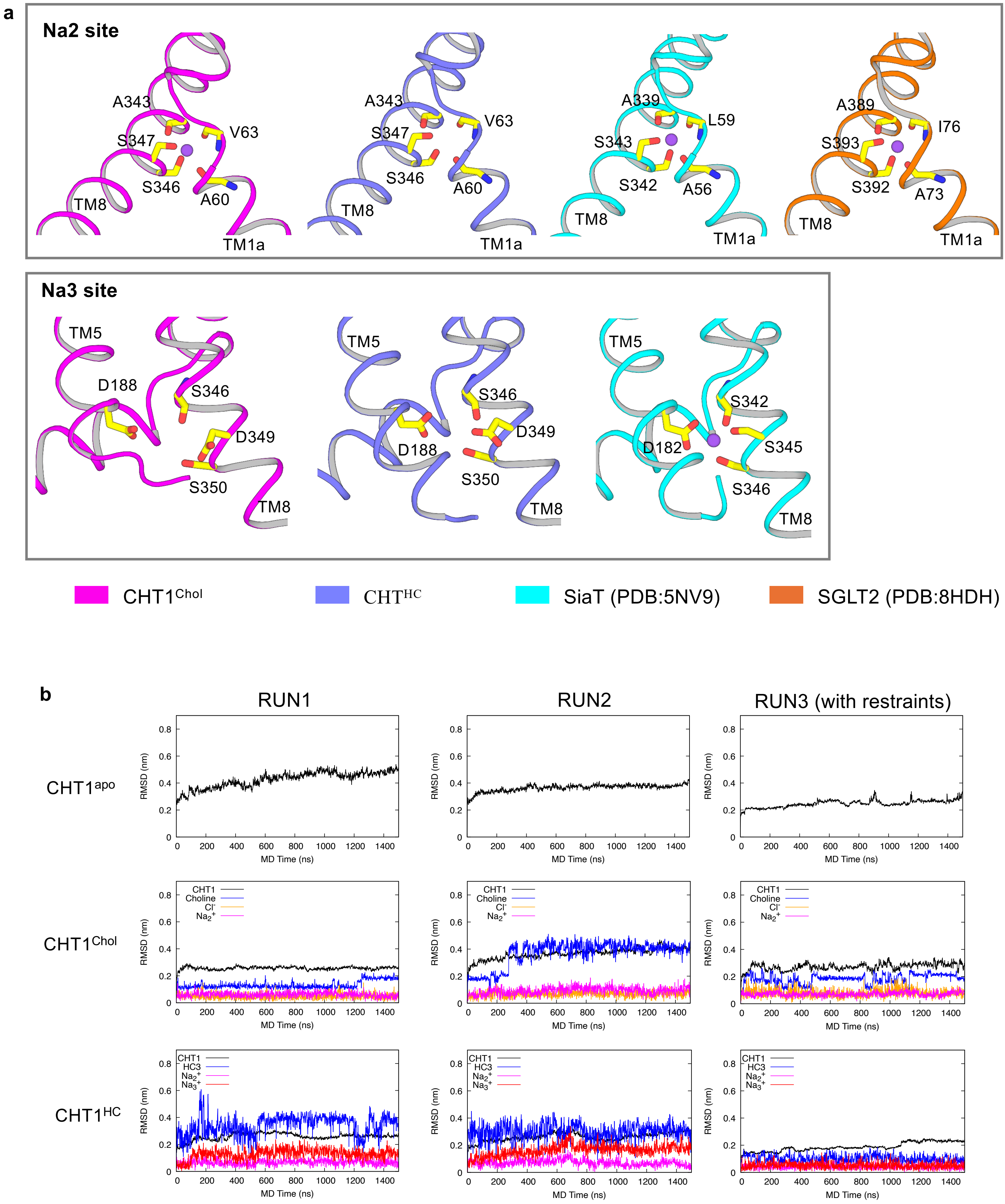
a
Na2 site
A343
S347
V63
S346
A60
TM8
TM1a
A343
S347
V63
S346
A60
TM8
TM1a
A339
L59
S343
S342
A56
TM8
TM1a
A389
I76
S393
S392
A73
TM8
TM1a
Na3 site
TM5
D188
S346
D349
S350
TM8
TM5
S346
D349
D188
S350
TM8
TM5
S342
D182
S345
S346
TM8
CHT1Chol
CHTHC
SiaT (PDB:5NV9)
SGLT2 (PDB:8HDH)
b
RUN1
RUN2
RUN3 (with restraints)
CHT1apo
CHT1Chol
CHT1HC
RMSD (nm)
MD Time (ns)
CHT1
Choline
Cl-
Na2+
HC3
Na3+

**Fig. S8 Substrate binding and dynamics in CHT1.**

**a,** Local structures at the Na2 and Na3 sites in CHT1$^{Chol}$, CHT$^{HC}$, SiaT, and SGLT2. Purple spheres represent the bound $Na^+$ ions observed in the structures.

**b,** MD simulations of CHT1 in the apo, choline-bound, and HC3-bound states. Three independent 1500 ns simulations were conducted for each state, with one simulation (RUN3) applying backbone position restraints on CHT1 to maintain the cryo-EM structure. For CHT1$^{Chol}$, choline, $Cl^-$, and $Na^+$ at the Na2 site were initially positioned in their respective binding sites. For CHT1$^{HC}$, HC3, along with $Na^+$ at the Na2 and Na3 sites, were initially placed in their binding sites. The protein RMSD of CHT1 from the cryo-EM structures and the heavy atom RMSD of choline, $Cl^-$, and $Na^+$ from their initial positions were plotted over time. Low RMSD values indicate high structural stability and ligand binding.

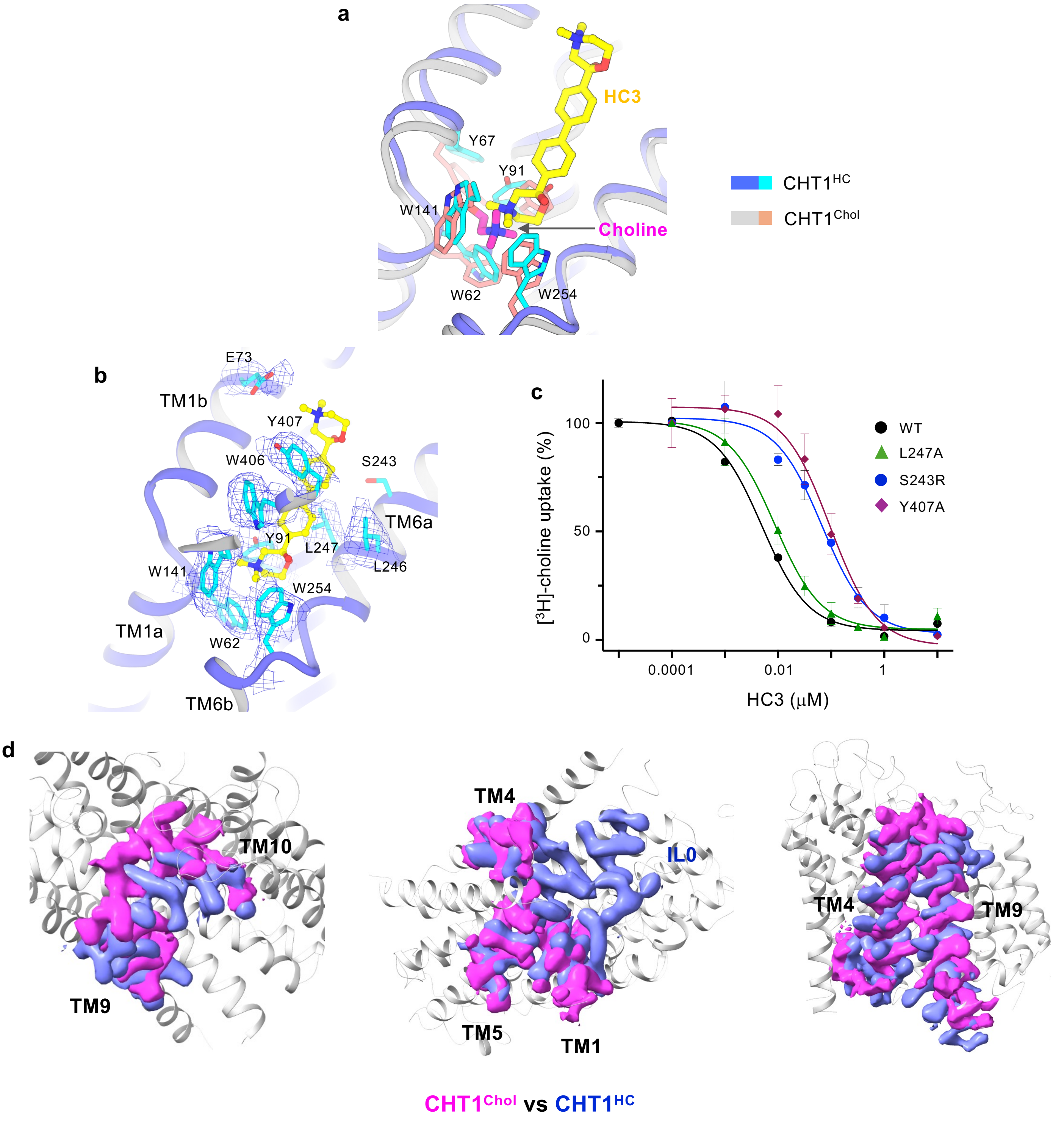


**Fig. S9 HC3 inhibition and conformational changes in CHT1$^{HC}$.**

**a,** Structural comparison of the choline-binding and HC3-binding pockets between CHT1$^{Chol}$ and CHT1$^{HC}$.

**b,** Cryo-EM density map of HC3 coordinating residues, as shown in Fig. 5b. The density (blue mesh) is contoured at 4 σ.

**c,** Concentration-dependent inhibition of choline uptake by HC3 for CHT1 WT and mutants. Data points are mean ± SEM (n = 3 independent experiments) and fitted to the three-parameter dose-response curves (GraphPad Prism 9) with IC50 of 4.98±1.04 nM for WT, 8.75±4.1 nM for L247A, 95.7±55.9 nM for Y407A and 67.4±32.8 nM for S243R.

**d,** Map densities of the regions undergoing conformational changes between CHT1$^{Chol}$ vs CHT1$^{HC}$, as shown in Fig. 5d-f.

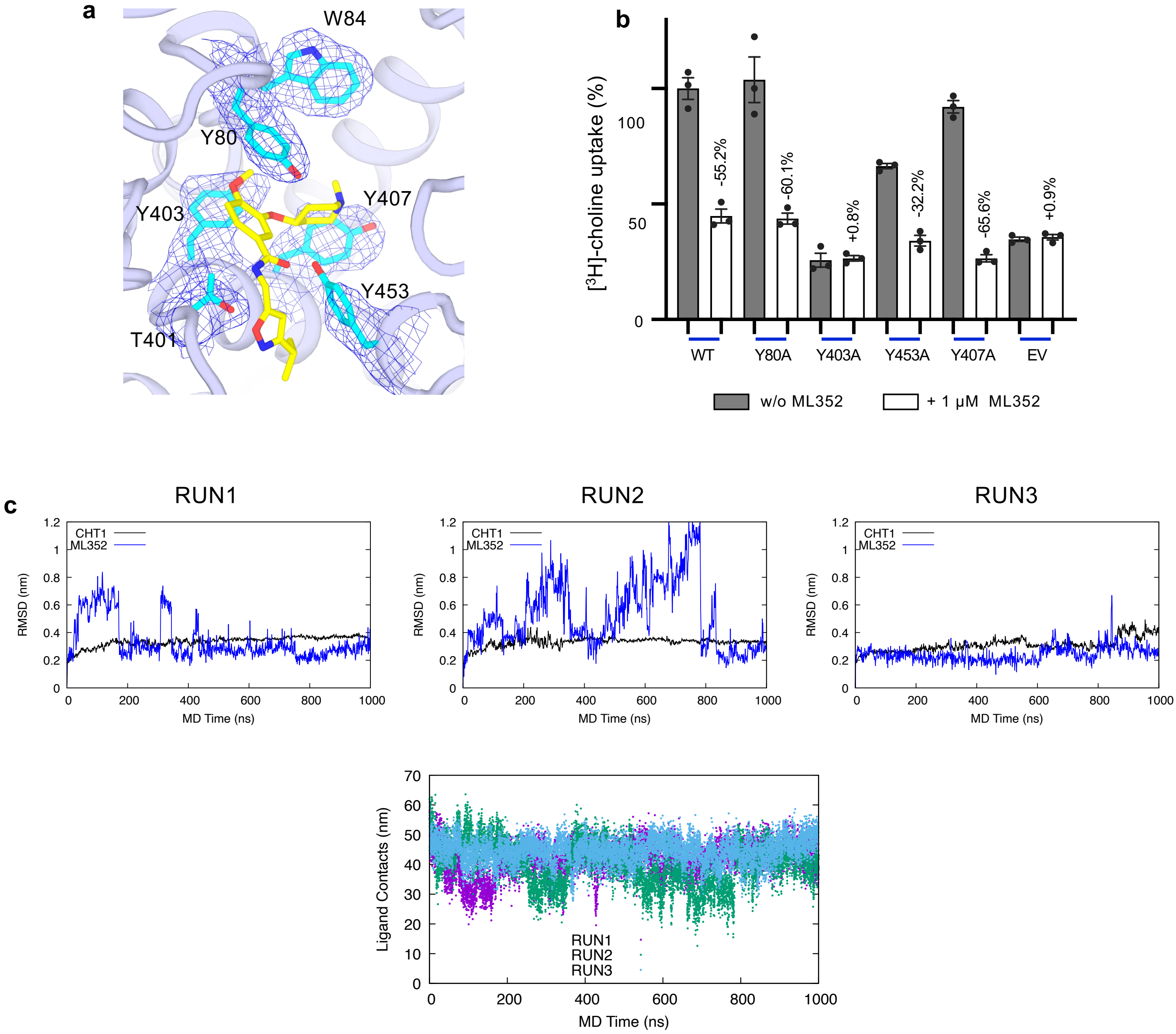


**Fig. S10 ML352 inhibition of CHT1.**

**a,** Cryo-EM density map of ML352 coordinating residues, as shown in Fig. 6c. The density (blue mesh) is contoured at 5 σ.

**b,** Radioactive choline uptake and inhibition for the CHT1 WT and variants with substitutions in the ML352-binding pocket. Difference between means before and after adding 1 μM ML352 is shown as percentage. Data points are mean ± SEM (n = 3 independent experiments).

**c,** MD simulations of CHT1 in the ML352-bound state were conducted with three independent 1000 ns unrestrained runs. The RMSD of ML352 from its initial position (upper panel) and the ligand contacts between ML352 and CHT1 (lower panel) were plotted over time. Despite some rotational dynamics, ML352 maintained stable binding within the proposed pocket, exhibiting extensive contacts with CHT1 throughout the simulations.

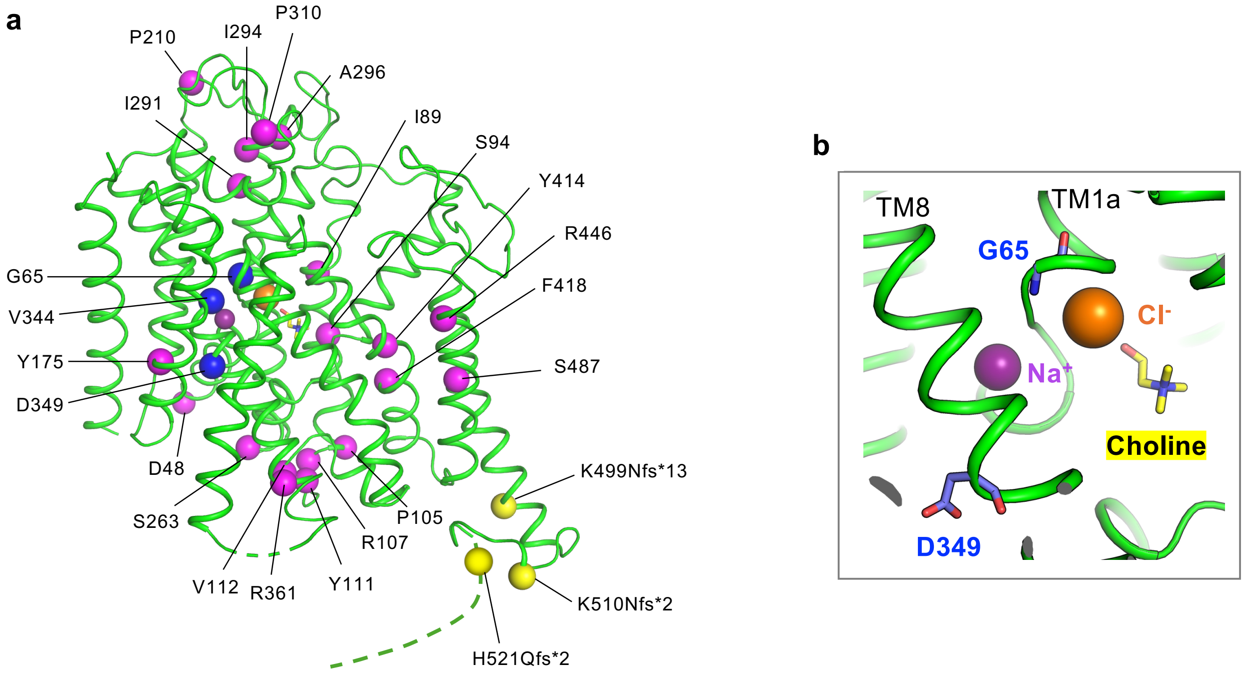


**Fig. S11 Structural mapping of disease-related mutations.**

**a,** Disease-related mutations mapped onto the CHT1 structure. Magenta, blue, and yellow spheres represent cell surface mutations, mutations within the CHT1 core involved in substrates binding, and frameshift mutations leading to C-terminal truncation, respectively.

**b,** Close-up view of the G65 and D349 mutations surrounding the substrates binding pocket.

## Table S1 Cryo-EM data collection, processing, and refinement statistics

| Structure | CHT1$^{apo}$ | CHT1$^{Chol}$ | CHT1$^{HC}$ | CHT1$^{ML352}$ |
|---|---|---|---|---|
| EMDB | EMD-44497 | EMD-44498 | EMD-44593 | EMD-44499 |
| PDB | 9BFI | 9BFJ | 9BIM | 9BFK |
| **Data collection/ processing** | | | | |
| Magnification | 165k | 165k | 105k | 165k |
| Voltage (kV) | 300 | 300 | 300 | 300 |
| Pixel size (Å) | 0.738 | 0.738 | 0.84 | 0.738 |
| Defocus range (μm) | -0.8 ~ -1.8 | -0.8 ~ -1.8 | -1.0 ~ -2.0 | -0.8 ~ -1.8 |
| Electron exposure ($e^-$/Å$^2$) | 60 | 60 | 60 | 60 |
| Symmetry imposed | C1 | C1 | C1 | C1 |
| Initial particles (No.) | 4,785,338 | 4,175,806 | 5,035,469 | 4,725,107 |
| Final particles (No.) | 334,300 | 452,081 | 191,427 | 317,376 |
| Map resolution (Å) | 2.66 | 2.35 | 3.67 | 2.85 |
| FSC threshold | 0.143 | 0.143 | 0.143 | 0.143 |
| Map sharpening B-factor (Å$^2$) | 113.1 | 86.3 | 177.0 | 126.9 |
| **Refinement** | | | | |
| Initial model used | AlphaFold: AF-Q9GZV3-F1 | | | |
| Model Resolution (Å) | 2.89 | 2.55 | 3.83 | 3.08 |
| FSC threshold | 0.5 | 0.5 | 0.5 | 0.5 |
| Model composition | | | | |
| Non-hydrogen atoms | 3,766 | 3,833 | 3,992 | 3829 |
| Protein residues | 489 | 496 | 516 | 494 |
| Ligands | 0 | 3 | 1 | 1 |
| | – | Choline, 1 | hemicholinium-3, 1 | ML352, 1 |
| | – | Na, 1 | – | – |
| | – | Cl, 1 | – | – |
| *B* factors (Å$^2$) | | | | |
| Protein (min/max/mean) | 28.78/116.59/58.2 | 24.95/113.48/52.8 | 33.71/147.24/57.64 | 32.17/135.05/65.22 |
| Ligand | – | 30.07/41.90/39.92 | 33.71/52.86/46.20 | 56.21/56.21/56.21 |
| R.m.s. deviations | | | | |
| Bond lengths (Å) | 0.003 | 0.005 | 0.004 | 0.003 |
| Bond angles (°) | 0.682 | 0.654 | 0.745 | 0.650 |
| **Validation** | | | | |
| MolProbity score | 1.05 | 1.12 | 1.42 | 1.14 |
| Clashscore | 2.64 | 3.24 | 4.86 | 3.52 |
| Rotamer outliers (%) | 0 | 0 | 0 | 0 |
| Ramachandran plot (%) | | | | |
| Favored | 98.54 | 98.98 | 97.07 | 98.77 |
| Allowed | 1.46 | 1.02 | 2.93 | 1.23 |
| Outliers | 0 | 0 | 0 | 0 |

**Supplementary Movies:**

**Movie S1:** Local structures at the ion- and substrate-binding sites of $CHT1^{Chol}$.

**Movie S2:** Conformational changes between $CHT1^{apo}$ and $CHT1^{Chol}$.

**Movie S3:** Conformational changes between $CHT1^{HC}$ and $CHT1^{Chol}$.

**Movie S4:** Working model for the $Na^+$- and $Cl^-$-dependent choline transport in CHT1.
